# Dihydrotanshinone, a Natural Diterpenoid, Preserves Blood-Retinal Barrier Integrity via P2X7 Receptor

**DOI:** 10.3390/ijms21239305

**Published:** 2020-12-06

**Authors:** Claudia Giuseppina Fresta, Giuseppe Caruso, Annamaria Fidilio, Chiara Bianca Maria Platania, Nicolò Musso, Filippo Caraci, Filippo Drago, Claudio Bucolo

**Affiliations:** 1 Department of Biomedical and Biotechnological Sciences, School of Medicine, University of Catania, 95125 Catania, Italy; forclaudiafresta@gmail.com (C.G.F.); chiara.platania@unict.it (C.B.M.P.); nmusso@unict.it (N.M.); fdrago@unict.it (F.D.); 2 Department of Drug Sciences, University of Catania, 95125 Catania, Italy; forgiuseppecaruso@gmail.com (G.C.); annafidilio@yahoo.it (A.F.); carafil@hotmail.com (F.C.); 3 Oasi Research Institute—IRCCS, 94018 Troina, Italy; 4 Center for Research in Ocular Pharmacology-CERFO, University of Catania, 95125 Catania, Italy

**Keywords:** diabetic retinopathy, blood-retinal barrier, purinergic P2X7 receptor, endothelial cells, oxidative stress, inflammation

## Abstract

Activation of P2X7 signaling, due to high glucose levels, leads to blood retinal barrier (BRB) breakdown, which is a hallmark of diabetic retinopathy (DR). Furthermore, several studies report that high glucose (HG) conditions and the related activation of the P2X7 receptor (P2X7R) lead to the over-expression of pro-inflammatory markers. In order to identify novel P2X7R antagonists, we carried out virtual screening on a focused compound dataset, including indole derivatives and natural compounds such as caffeic acid phenethyl ester derivatives, flavonoids, and diterpenoids. Molecular Mechanics/Generalized Born Surface Area (MM/GBSA) rescoring and structural fingerprint clustering of docking poses from virtual screening highlighted that the diterpenoid dihydrotanshinone (DHTS) clustered with the well-known P2X7R antagonist JNJ47965567. A human-based in vitro BRB model made of retinal pericytes, astrocytes, and endothelial cells was used to assess the potential protective effect of DHTS against HG and 2′(3′)-O-(4-Benzoylbenzoyl)adenosine-5′-triphosphate (BzATP), a P2X7R agonist, insult. We found that HG/BzATP exposure generated BRB breakdown by enhancing barrier permeability (trans-endothelial electrical resistance (TEER)) and reducing the levels of ZO-1 and VE-cadherin junction proteins as well as of the Cx-43 mRNA expression levels. Furthermore, HG levels and P2X7R agonist treatment led to increased expression of pro-inflammatory mediators (TLR-4, IL-1β, IL-6, TNF-α, and IL-8) and other molecular markers (P2X7R, VEGF-A, and ICAM-1), along with enhanced production of reactive oxygen species. Treatment with DHTS preserved the BRB integrity from HG/BzATP damage. The protective effects of DHTS were also compared to the validated P2X7R antagonist, JNJ47965567. In conclusion, we provided new findings pointing out the therapeutic potential of DHTS, which is an inhibitor of P2X7R, in terms of preventing and/or counteracting the BRB dysfunctions elicited by HG conditions.

## 1. Introduction

Diabetic retinopathy (DR) is one of the microvascular complications of diabetes. DR is a leading cause of vision impairment among the working-age population [1]. It has been estimated that the DR incidence will grow up, affecting 190 million patients by 2030, by pointing out the importance of research efforts toward new diagnostic and therapeutic strategies [2]. DR progresses from a non-proliferative (NPDR) stage to a proliferative (PDR) stage, this latter is characterized by vitreous hemorrhages and extensive neovascularization [3]. Furthermore, DR in early stages is characterized by pericytes and endothelial cells death [4]. Indeed, changes in the vascular endothelial membrane as well as vascular leakage compromise the blood-retinal barrier (BRB) [5], whose structural integrity is essential for retinal homeostasis and function [6]. In fact, the loss of the BRB integrity significantly contributes to the pathophysiology of several retinal disorders including DR [7]. The BRB is a tight and limitative barrier that manages the flux of ions, proteins, metabolic waste compounds, and water flow through the retina, and consists of two distinct regions including the inner and outer BRB [8]. The outer BRB (oBRB) is formed by retinal pigmented epithelial cells connected by tight junction proteins, while the inner BRB (iBRB) is established by tight junctions between retinal capillary endothelial cells, surrounded by pericytes and supported by glial cells [9]. Among glial cells, astrocytes provide functional support to the iBRB, playing a crucial role for the maintenance of retinal endothelial capillaries’ integrity [10].

Zonula occludens-1, -2, and -3 (ZO-1 ZO-2, and ZO-3), occludins, and claudins are tight junctions essential for BRB structural stability [9]. A decrease of the tight junction protein expression levels, and the consequent BRB breakdown, has been shown in experimental models of diabetes [11]. According to Osanai et al., tight junction proteins are involved in many physiological processes including cell proliferation and differentiation. Furthermore, they limit the passage of proteins and lipids between apical and basolateral membranes, guiding endothelial cell polarity [12]. The maintenance of the BRB integrity is also due to the interactions between tight and adherens junctions, mediated by cell-cell adhesion molecules such as cadherins [13], which represent a family of proteins implicated in the maintenance of endothelial cells’ adhesion to the vasculatures, including VE-cadherin [14]. Since several studies have shown the role played by the inflammatory processes in diabetes-associated retinal alterations, the interest on the link between reactive oxygen species (ROS), inflammatory processes, and endothelial dysfunction has been increasing. The inflammatory processes occurring during the development of DR lead to the activation of toll-like receptors 4 (TLR-4) that, in turn, trigger the overexpression of pro-inflammatory cytokines and acute phase proteins [15]. In particular, IL-1β and TNF-α are involved in the pathogenesis of DR, concurring to diabetes-induced degeneration of retinal capillaries [16,17]. Still in the context of inflammation, an additional pathophysiological event is the interaction between leucocytes and endothelial cells. Indeed, this cell interaction is fundamental for the recruitment of leucocytes at the inflammation site [18]. In particular, intercellular adhesion molecule-1 (ICAM-1), whose expression in the retina is increased in DR [19], has been linked to both leukostasis and inflammatory phenomena [20].

During the last three decades, several studies have demonstrated the pivotal role played by ROS in retinal microvascular complications, such as DR [21]. Since the retina is a high energy-demanding organ, it becomes more susceptible to high levels of ROS, that, along with a hyperglycemic environment, enhance mitochondrial dysfunction, inflammation, and degeneration pathways, leading to vascular, neural, and retinal tissue damage via pyroptosis and/or apoptosis [22].

Inside the retina, the combination between hyperglycemic and hypoxic conditions represents a strong stimulus for both astrocytes and endothelial cells, which, therefore, enhance the expression of a vascular endothelial growth factor (VEGF) [23]. When hyperglycemia becomes chronic, VEGF deflects from its physiological functions, leading to the formation of abnormal new blood vessels as observed for PDR [24]. Furthermore, it has been shown that this trophic factor, by interacting with the VEGF receptor 2, promotes endothelial tight-junctions’ alteration and development of endothelial cell fenestration, resulting in a thinning of the blood vessels [25].

Endothelial and pericyte dysfunction represents a key factor for DR progression and increased extracellular ATP (eATP) that has been observed at the site of inflammation as a consequence of endothelial cell injury [26]. Moreover, it is well-established that the hyperglycemic environment leads to an enhanced eATP concentration, that triggers a cascade of events culminating in the activation of the purinergic signaling pathway, which includes the P2X7 receptor (P2X7R) [27]. In literature, there are numerous results highlighting the role of this ATP-gated ion channel in modulating inflammatory responses in the retinal microvasculature [28,29,30]. Studies conducted in experimental models of DR revealed that the P2X7R mediates the vascular inflammatory reactions due to an overexpression of cytokines, thus, contributing to BRB dysfunction, ischemia, and retinal vascular occlusion [29,31]. When considering endothelial cells, it has been recently demonstrated that high glucose (HG) conditions led to P2X7R over-activation in these cells, increased expression of pro-inflammatory markers, decreased cell viability, and, finally, loss of BRB integrity [32].

In the present work, we aimed to explore the role played by the P2X7R in a recently established in vitro primary culture based on triple co-culture BRB model entirely based on human cells (retinal perycites, retinal astrocytes, and retinal endothelial cells) [33]. Along with specific hallmarks of P2X7R activation in the iBRB (barrier permeability and expression of IL-1β, Cx43, tight and adherens junctions), we evaluated the effects of P2X7R inhibition on expression of other inflammatory cytokines, including VEGF, IL-6, IL-8, and TNF-α, along with ROS production in order to estimate an expression network linked to P2X7 signalling. We also investigated the potential protective effect of dihydrotanshinone (DHTS), a natural diterpenoid extracted from *Salvia miltiorrhiza*, against the retinal damage elicited by HG and a selective P2X7R agonist [2′(3′)-O-(4-Benzoylbenzoyl)adenosine-5′-triphosphate (BzATP)].

## 2. Results

### 2.1. Virtual Screening at P2X7R

**Figure 1:**
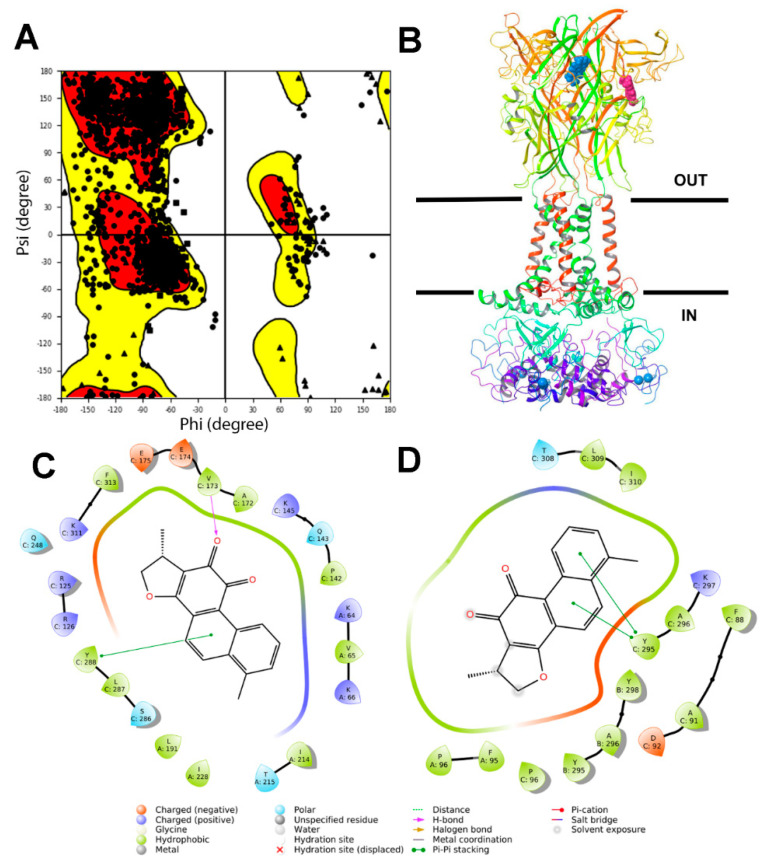
DHTS is predicted to bind the allosteric site of the human P2X7R. (**A**) Ramachandran plot of the full-length hP2X7R model, after energy minimization in an implicit solvent and membrane. (**B**) Representation of DHTS poses in the orthosteric (magenta Van der Waals spheres) and the allosteric (blue Van der Waals spheres) pockets of P2X7R. (**C**) The ligand interaction of a 2D diagram of DHTS in the orthosteric pocket of P2X7R. (**D**) The ligand interaction 2D diagram of DHTS in the allosteric pocket of P2X7R.

According to the last solved structure of a full-length rat P2X7R [34], we built the human full-length P2X7R model and minimized it in an implicit membrane model (Figure 1).

A series of compounds [35] have been screened and the database included known P2X7R allosteric antagonists [36]. Structural fingerprint clustering was carried out and DHTS, along with quercetin, clustered with JNJ47965567, which is a validated P2X7R allosteric inhibitor (Table 1).

Furthermore, we carried out molecular docking and Molecular Mechanics/Generalized Born Surface Area (MM/GBSA) calculations of DHTS binding at P2X7R allosteric, orthosteric pockets. We included in our analysis a pocket in the cytosolic domain of P2X7R, as identified by the SiteMap^®^ task of Schrodinger Maestro. According to our computational analysis (Table 1), DHTS can be an allosteric P2X7R antagonist, given the lowest (more favorable) predicted binding free energy at the allosteric pocket, compared to the orthosteric one. In this perspective, we carried out in vitro studies on retinal endothelial cells in order to assess the DHTS activity as a P2X7R antagonist, by treating cells with this putative antagonist, HG, and the selective P2X7R agonist BzATP.

### 2.2. BRB Integrity

**Figure 2:**
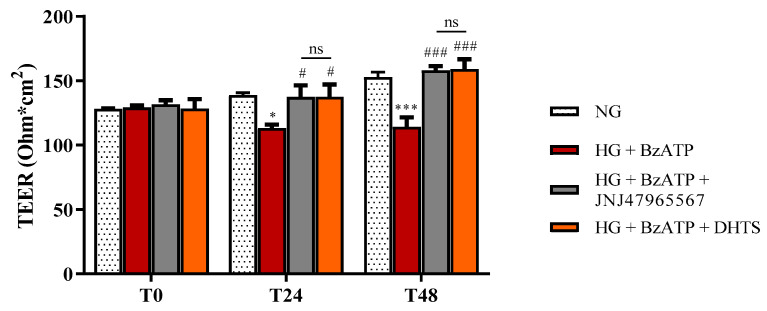
Assessment of barrier integrity in the in vitro human primary culture based on triple co-culture iBRB model by TEER under our experimental conditions. TEER values were measured at time 0 (T0), and after 24 (T24) and 48 (T48) h. NG = normal glucose condition (5 mM). HG = high glucose condition (40 mM). BzATP = 200 µM. JNJ47965567 = 100 nM. DHTS = 500 nM. Values are reported as means ± standard deviation (SD) of three independent experiments. Statistical analysis was performed using two-way analysis of variance (ANOVA) with Tukey’s *post-hoc* analysis. * *p* < 0.05 vs. NG. *** *p* < 0.001 vs. NG. ^#^ *p* < 0.05 vs. HG + BzATP. ^###^ *p* < 0.001 vs. HG + BzATP. ns = not significant.

iBRB integrity was evaluated by performing trans-endothelial electrical resistance (TEER) measurements and the paracellular permeability assay, following an exposure for 24 or 48 h to HG levels and BzATP (200 µM) in the absence or in the presence of JNJ47965567 (100 nM) or DHTS (500 nM) (2 h pre-treatment). Co-stimulus with HG and BzATP led to significant, albeit small, decreased TEER values, −18.5%, at 24 h, that further increased to −25.3% at 48 h, i.e., an increased permeability of our triple co-culture model of iBRB (Figure 2). DHTS and JNJ47965567 (validated, P2X7R antagonist) pre-treatment separately prevented the alterations of iBRB integrity (Figure 2).

**Figure 3:**
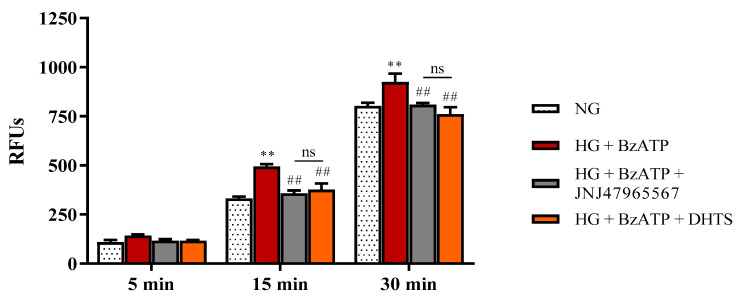
Measurement of apical-to-basolateral Na-F permeability in our iBRB model (in vitro human primary culture based on triple co-culture). Na-F permeability was measured after 5, 15, and 30 min. NG = normal glucose condition (5 mM). HG = high glucose condition (40 mM). RFUs = relative fluorescence units. BzATP = 200 µM. JNJ47965567 = 100 nM. DHTS = 500 nM. Values are reported as means ± SD of three independent experiments. Statistical analysis was performed using two-way ANOVA with Tukey’s *post-hoc* analysis. ** *p* < 0.01 vs. NG. ^##^ *p* < 0.01 vs. HG + BzATP. ns = not significant.

These data were also confirmed by measuring the apical-to-basolateral permeability of sodium fluorescein (Na-F). Na-F permeability accounts to paracellular permeability across the endothelial cells/perycites monolayers under our experimental conditions [33] (Figure 3).

HG/BzATP stimulus significantly increased the Na-F fluorescence, indicating that HG and activation of P2X7R led to higher permeability of endothelial cells/perycites monolayers (*p* < 0.05 vs. NG), confirming the TEER measurements. The inhibition of P2X7R by pre-treatment with JNJ47965567 significantly decreased the Na-F permeability, counteracting the effects of HG + BzATP. According to virtual screening predictions, DHTS (putative P2X7R antagonist) also decreased the monolayer Na-F permeability, preventing and/or counteracting the damage induced by both HG and P2X7R agonist (BzATP) as already shown in TEER measurements. Therefore, JNJ47965567 and DHTS exerted protective effects on iBRB integrity challenged with the co-stimuli HG and BzATP.

### 2.3. Junctional Proteins

**Figure 4:**
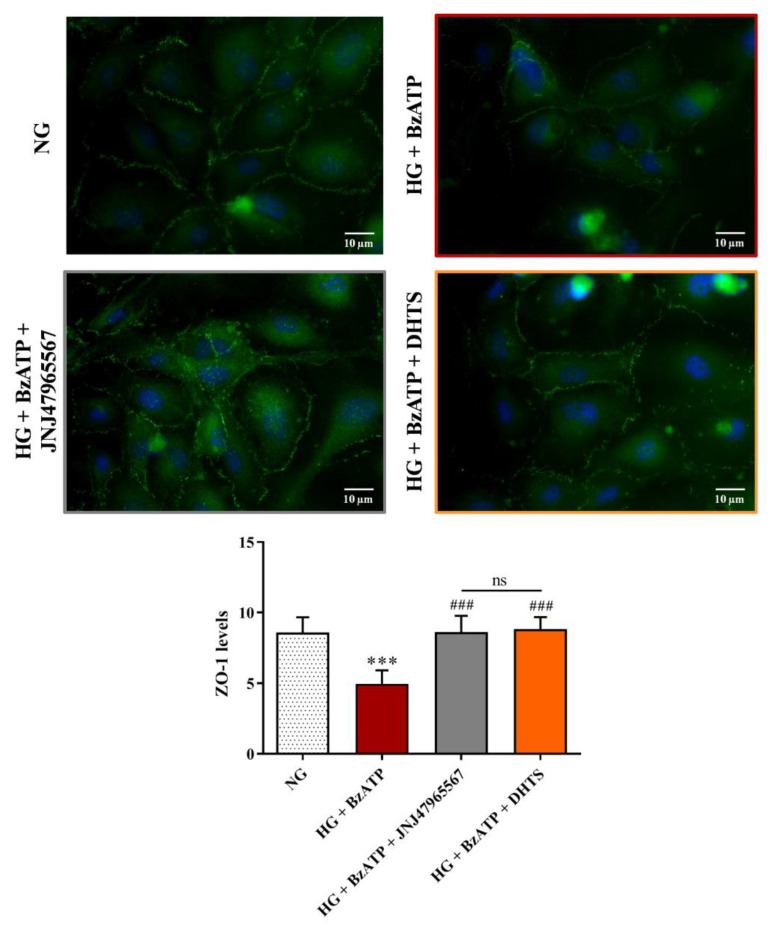
Immunocytochemistry evaluation of ZO-1 staining in endothelial cells subjected to normal or high glucose conditions + BzATP, in the absence or in the presence of JNJ47965567 or DHTS, for 48 h. ZO-1 was labeled with FITC (green) while nuclei were labeled with DAPI (blue). Images for ZO-1 immunostaining were acquired at 40× magnification. Scale bar: 10 µm. NG = normal glucose condition (5 mM). HG = high glucose condition (40 mM). BzATP = 200 µM. JNJ47965567 = 100 nM. DHTS = 500 nM. The average intensity (AU) of the data from more than 30 cells per coverslip for ZO-1 under our experimental conditions are shown. Values are reported as means ± SD of three independent experiments. Statistical analysis was performed using one-way ANOVA with Tukey’s *post-hoc* analysis. *** *p* < 0.001 vs. NG. ^###^ *p* < 0.001 vs. HG + BzATP. ns = not significant.

Since iBRB integrity is dependent on the expression of tight (ZO-1, ZO-2, and ZO-3, occludins, and claudins) and adherens junctions, we carried out immunocytochemistry experiments to assess the expression of ZO-1 and VE-cadherin proteins in endothelial cells’ monolayer, which is part of our iBRB model. As shown in Figure 4, ZO-1 expression, measured as fluorescence arbitrary units (AUs), was significantly reduced after an exposure for 48 h to HG in combination with BzATP compared to NG conditions, whereas JNJ47965567 or DHTS pre-treatment protected against the reduction of ZO-1 expression HG/BzATP-induced, preserving and/or counteracting the continuous brush border observed in control levels (Figure 4).

**Figure 5:**
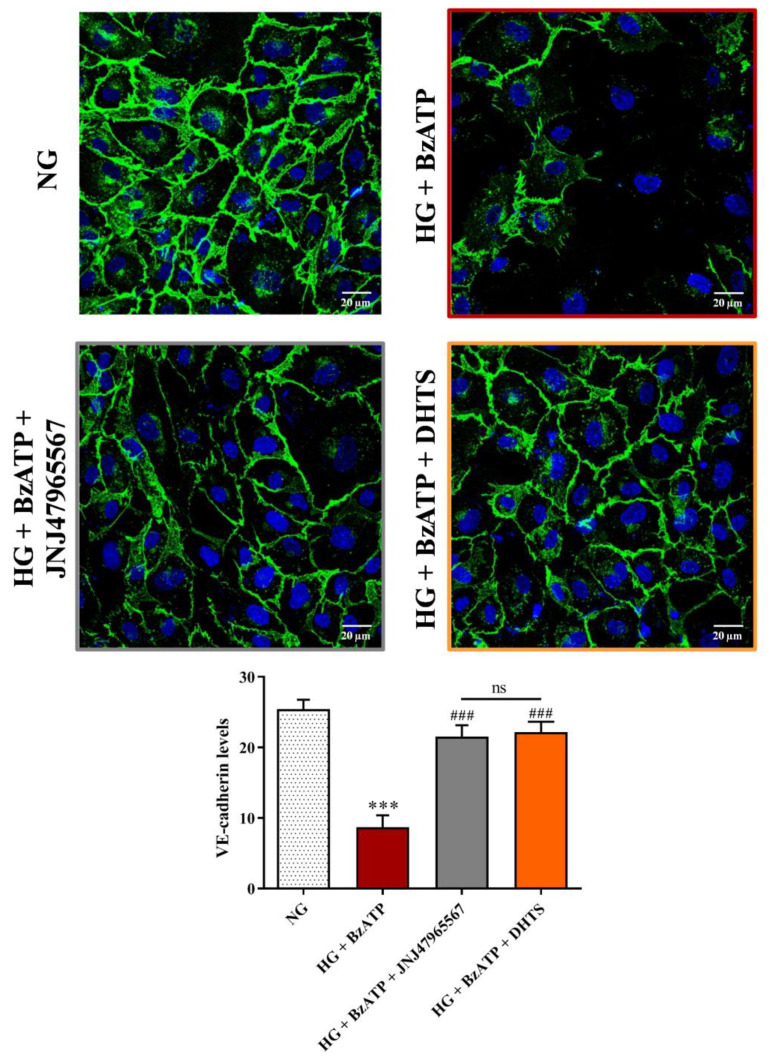
Confocal analysis of VE-cadherin in endothelial cells subjected to normal or high glucose conditions + BzATP, in the absence or in the presence of JNJ47965567 or DHTS, for 48 h. VE-cadherin was labeled with FITC (green) while nuclei were labeled with DAPI (blue). Images for VE-cadherin immunostaining were acquired at 20× magnification. Scale bar: 20 µm. NG = normal glucose condition (5 mM). HG = high glucose condition (40 mM). BzATP = 200 µM. JNJ47965567 = 100 nM. DHTS = 500 nM. The average intensity (AU) of the data from more than 30 cells per coverslip for VE-cadherin under our experimental conditions are shown. Values are reported as means ± SD of three independent experiments. Statistical analysis was performed using one-way ANOVA with Tukey’s *post-hoc* analysis. *** *p* < 0.001 vs. NG. ^###^ *p* < 0.001 vs. HG + BzATP. ns = not significant.

A similar trend was observed for expression of the adherens junction VE-cadherin. Figure 5 shows a significant reduction of VE-cadherin staining at the cell-cell contacts following the exposure to the HG/BzATP insult, as compared to cells grown in NG conditions. On the other hand, an increased expression of VE-cadherin was observed at an endothelial cell-cell interface after the pre-treatment with JNJ47965567 or DHTS compounds (Figure 5).

### 2.4. ROS Production

**Figure 6:**
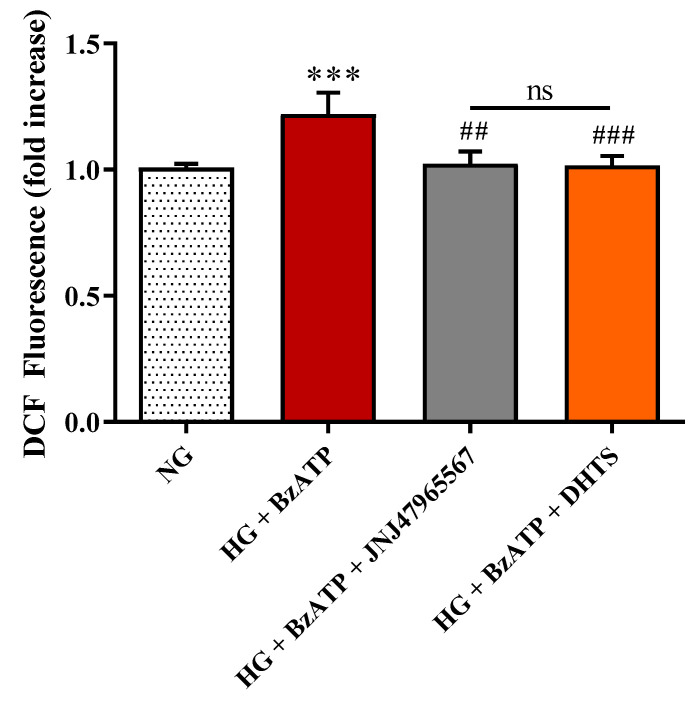
Intracellular ROS production in endothelial cells under our experimental conditions. NG = normal glucose condition (5 mM). HG = high glucose condition (40 mM). BzATP = 200 µM. JNJ47965567 = 100 nM. DHTS = 500 nM. Values are reported as means ± SD of three independent experiments. Statistical analysis was performed using one-way ANOVA with Tukey’s *post-hoc* analysis. *** *p* < 0.001 vs. NG. ^##^ *p* < 0.01 vs. HG + BzATP. ^###^ *p* < 0.001 vs. HG + BzATP. ns = not significant.

In order to investigate the role played by P2X7R signaling in HG-induced oxidative stress in endothelial cells, part of the in vitro tri-culture BRB model, we employed the ROS sensitive dye 2′,7′-dichlorofluorescin diacetate (DCFDA). After stimulation for 48 h with HG + BzATP, there was a significant (*p* < 0.001) increase in ROS formation compared to control conditions. JNJ47965567 or DHTS pre-treatment significantly prevented the increase in ROS production elicited by HG + BzATP (Figure 6).

### 2.5. Inflammatory Biomarkers

It is well-established that endothelial dysfunction is linked to P2X7R over-activation [27] and pro-inflammatory phenomena, culminating in the development of DR [26]. The vascular inflammation P2X7R-mediated is due, among other things, to the unregulated production of cytokines, strongly contributing to BRB dysfunction, ischemia, and retinal vascular occlusion [29,31]. By using an endothelial cell monolayer, we have recently demonstrated that HG conditions led to P2X7R over-activation, paralleled by increased expression of pro-inflammatory markers and decreased cell viability [32].

**Figure 7:**
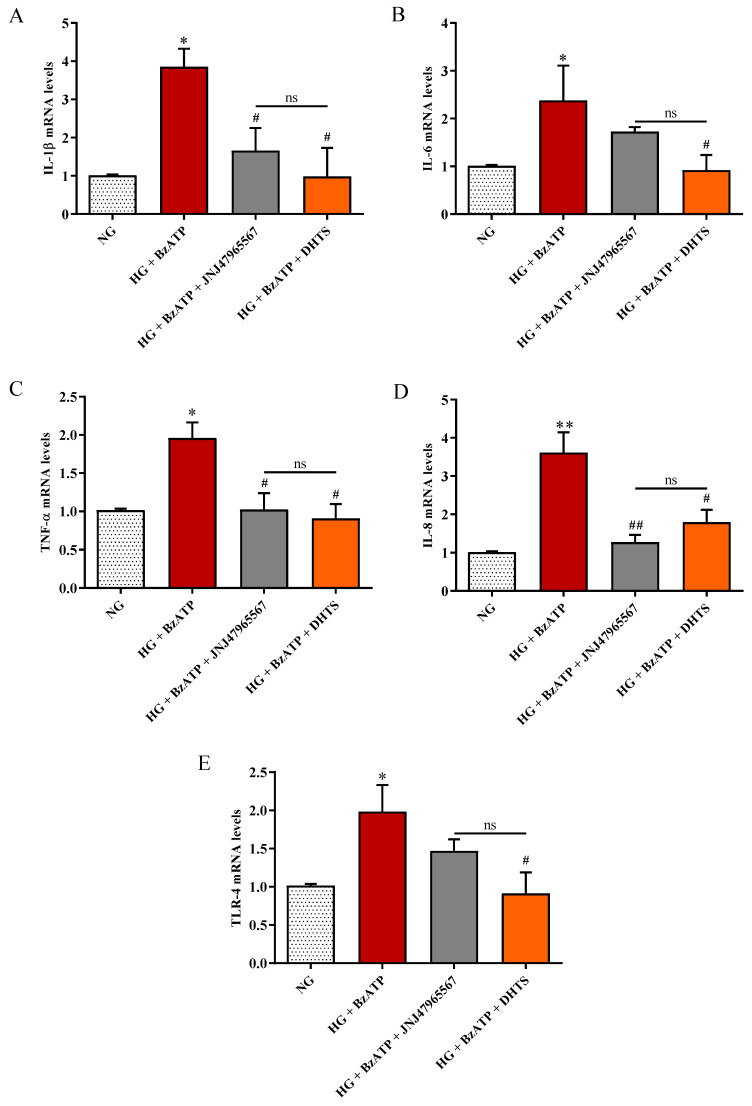
Measurement of (**A**) IL-1β, (**B**) IL-6, (**C**) TNF-α, (**D**) IL-8, and (**E**) TLR-4 mRNA levels (quantitative real-time PCR (qRT-PCR)) in endothelial cells under our experimental conditions. NG = normal glucose condition (5 mM). HG = high glucose condition (40 mM). BzATP = 200 µM. JNJ47965567 = 100 nM. DHTS = 500 nM. The abundance of each mRNA of interest was expressed relatively to the abundance of 18S rRNA, as an internal control. Values are reported as means ± SD of three independent experiments. Statistical analysis was performed using one-way ANOVA with Tukey’s *post-hoc* analysis. ** *p* < 0.01 vs. NG. * *p* < 0.05 vs. NG. ^##^ *p* < 0.01 vs. HG + BzATP. ^#^ *p* < 0.05 vs. HG + BzATP. ns = not significant.

As shown in Figure 7A–D, the combined treatment with HG and BzATP led to the significant over-expression at the mRNA level of IL-1β, IL-6, TNF-α, and IL-8, which are the major mediators of inflammation-induced damage, in retinal endothelial cells compared to NG conditions (*p* < 0.01 for IL-8, *p* < 0.05 for the remaining cytokines).

Inhibition of P2X7R, through JNJ47965567 pre-treatment, significantly decreased cytokines levels such as IL-1β (*p* < 0.05), TNF-α (*p* < 0.05), and IL-8 (*p* < 0.01), counteracting the inflammatory stimuli exerted by HG + BzATP. The “predicted” P2X7R antagonist DHTS significantly decreased the expression levels of the analyzed pro-inflammatory markers (*p* < 0.05) to values of control cells. Furthermore, HG + BzATP stimulation led to a significant increased expression of TLR-4 expression compared to NG conditions (*p* < 0.05) (Figure 7E). DHTS pre-treatment significantly reduced TLR-4 expression levels compared to the HG + BzATP condition (*p* < 0.05 vs. HG + BzATP). As observed for IL-6, JNJ47965567 pre-treatment diminished, although not significantly, the mRNA levels of TLR-4 in endothelial cells challenged with HG + BzATP. The expression at the mRNA level of matrix metallopeptidase 9 (MMP-9), which is an additional pro-inflammatory marker, NADPH oxidase 2, which is a pro-oxidant enzyme, and transforming growth factor beta-1 (TGF-β1), which is an anti-inflammatory cytokine, was measured. We did not detect any significant changes for these three targets under our experimental conditions (data not shown).

### 2.6. P2X7R, Cx-43, VEFG-A, and ICAM-1 Expression

**Figure 8:**
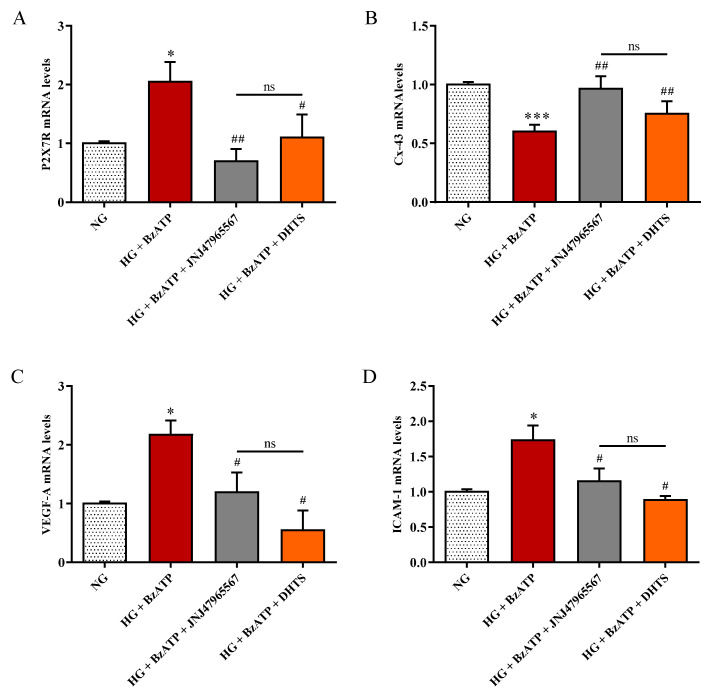
Measurement of (**A**) P2X7R, (**B**) Cx-43, (**C**) VEGF-A, and (**D**) ICAM-1 mRNA expression levels (qRT-PCR) in endothelial cells under our experimental conditions. NG = normal glucose condition (5 mM). HG = high glucose condition (40 mM). BzATP = 200 µM. JNJ47965567 = 100 nM. DHTS = 500 nM. The abundance of each mRNA of interest was expressed relatively to the abundance of 18S rRNA, as an internal control. Values are means ± SD of three independent experiments. * *p* < 0.05 vs. NG. *** *p* < 0.001 vs. NG. ^##^ *p* < 0.01 vs. HG + BzATP. ^#^ *p* < 0.05 vs. HG + BzATP. ns = not significant.

In order to assess the effects of HG and P2X7R activation on the expression of connexin-43 (Cx-43) and P2X7R, we carried out qRT-PCR experiments. Furthermore, accordingly to pathophysiology markers of DR, we included the study of VEGF-A and ICAM-1 mRNA levels under our experimental conditions in our analysis assessment (Figure 8). The treatment with HG + BzATP significantly raised P2X7R, VEFG-A, and ICAM-1 mRNA expression levels in endothelial cells when compared to NG conditions (*p* < 0.05), while the opposite effect was observed for Cx-43 (*p* < 0.001). JNJ47965567 or DHTS pre-treatment significantly counteracted the over-expression of P2X7R, VEGF-A, and ICAM-1 induced by HG + BzATP treatment, and rescued Cx-43 expression levels to values of control cells (NG condition).

## 3. Discussion

BRB dysfunction represents a well-known hallmark of DR [37]. However, the molecular mechanisms underlying this damage are not fully elucidated yet. To shed more light on these mechanisms, we employed our recently developed BRB triple co-culture model [33]. An alternative BRB triple co-culture model was previously developed by Wisniewska-Kruk and colleagues in which bovine endothelial cells and pericytes as well as rat astrocytes were used to study the effects of VEGF stimulation [38]. Despite the substantial contribution to the understanding of BRB dysfunction, it is worth noting that the authors employed a mixed (bovine/rat) system, which is a bit far from human BRB. Our system used only human cells with the same in vivo cellular numerical ratio, mimicking the human milieu. However, there are other innovate systems such as BRB-on-chip trying to mimic and control the microenvironment, even though no iBRB-on-chip based on human triple co-culture has been proposed so far [39,40].

During the last decade, the signaling pathway of P2X7R, which is an ATP-gated purinergic channel, has been associated with inflammatory phenomena at a retinal level [31]. Accordingly to the established liaison between inflammasome NLR family pyrin domain containing 3 (NLRP3) and P2X7R, channel activation is connected to release of mature inflammatory cytokines [41], after inflammatory priming linked to the activation of the receptor for an advanced glycation end product (RAGE) and signaling mediated by TLR-4. Therefore, various inflammatory cytokines (e.g., IL-1β and TNF-α) produced by different retinal cell types, such as endothelial cells, have been found to be increased in vitreous and aqueous humor from patients with the early stages of DR [42], strengthening the role of inflammation in disease progression [43]. In fact, before approval of anti-VEGF for treatment of diabetic macular edema, intravitreal steroids were considered the standard of care in DR treatment [44]. Previous preclinical studies highlighted the leading role of P2X7R in modulation of inflammation in in vitro and in vivo models of DR [30,36,45,46,47], along with the modulation of the expression of tight and adherens junctions in the endothelial monolayer [32]. P2X7R antagonists were reported to counteract inflammation in several animal and in vitro models of retinal diseases [48,49,50,51]. P2X7R antagonists were initially designed as anti-cancer agents, but did not reach the market due to clinical trial failures [52]. Currently, a selective P2X7 antagonist designed by Janssen is tested in a phase II clinical trial for the treatment of depression [53]. Searching for novel effective P2X7R antagonists, we carried out a virtual screening on a small in-house compound dataset, previously screened for discovery of Elav-1 (HuR) inhibitors [35,53]. Our virtual screening and MM/GBSA rescoring identified DHTS as a hit P2X7R antagonist. MM-GBSA calculations provide high correlation of binding energy vs. experimental activity of congeneric or non-congeneric series of compounds [54,55,56] and this computational approach was already validated for P2X7R allosteric antagonists. Although we did not provide single channel electrophysiological studies for characterization of DHTS activity at P2X7R, predicted binding free energy supported the hypothesis that DHTS would be a P2X7R allosteric antagonist. Therefore, we tested in vitro the activity of DHTS as a P2X7R antagonist in a triple culture model of iBRB exposed to the HG and BzATP (selective P2X7R agonist) challenge [32,57,58]. In this experimental paradigm, we included, as a positive P2X7R antagonist control, the validated P2X7R antagonist JNJ47965567. Since a well-defined BRB damage can be detected both at both 24 and 48 h [33,59], we challenged the BRB system with a HG/BzATP insult and evaluated the barrier integrity at these time points. As expected, the stimulation with HG/BzATP determined a significant decrease of TEER values after 24 h when compared to NG conditions, that further decreased after the 48 h challenge (Figure 2). The TEER results are correlated with the significant increased Na-F permeability through the BRB (Figure 3), indicating a dysfunction of the retinal barrier under hyperglycemic conditions, which is a clinical state often observed in DR patients that are not under strict glycemic control [60]. These results are also in accordance with previous findings showing a reduced retinal blood flow and disrupted vascular function in the diabetic retina due to P2X7R activation [31]. Of note, the DHTS treatment, similarly to JNJ47965567, during HG/BzATP stimulation, preserved the BRB integrity, according to TEER and Na-F data (Figure 2 and Figure 3). The protective potential of both molecules that antagonized HG/BzATP-induced BRB damage was also confirmed by the modulation of the expression of endothelial junctional proteins (Figure 4 and Figure 5), according to previous findings on endothelial mono-culture [32]. Reduced ZO-1 and VE-cadherin expression at the membrane cell-cell interface is one of the causes of BRB permeability in DR pathology. Therefore, P2X7R activity modulation by a selective antagonist would have an important translational impact [61,62,63,64,65].

Along with inflammation, another factor that contributes to DR progression is oxidative stress [66,67]. As shown by El-Remessy et al., increased oxidative stress due to the formation of different reactive species such as nitric oxide, superoxide, and their reaction product peroxynitrite, which significantly contributes to the diabetes-induced endothelial dysfunction and death [68]. Furthermore, Shibada et al. reported that the activation of P2X7R led to the retinal microvessels toxicity, inducing the overproduction of ROS [30]. It is also well-known that the progressive dysfunction of endothelial cells plays a pivotal role in BRB breakdown and other vascular alterations such as the loss of perivascular cells and dysregulated neovascularization [69]. However, despite that, molecular mechanisms leading to endothelial cells dysfunction during DR development are not completely understood yet and need to be further investigated. In consideration of the results showing the dysregulation of endothelial junction proteins after the HG/BzATP insult (Figure 4 and Figure 5) [32], we aimed to explore the gene expression network linked to P2X7R signaling in endothelial cells, which is part of the BRB model. Therefore, we analyzed mRNA levels of inflammatory cytokines, TLR- 4 receptor, VEGF-A, ICAM-1, P2X7R, and Cx-43 in endothelial cells pre-treated with DHTS, which is a predicted P2X7R antagonist. Regulation of gene expression in our system can be an indirect effect of P2X7R activity regulation, along with inflammasome recruitment and activation [41,70]. This hypothesis is supported by a previous study focused on VEGF expression, which was linked to inflammasome activation and purinergic signaling involving P2Y_2_ receptor [71]. We included a JNJ47965567 treatment group as a positive P2X7R antagonist control.

We also focused on the intracellular production of total ROS in endothelial cells, showing that their levels, significantly increased as a consequence of HG/BzATP treatment, were significantly decreased by the presence of DHTS or JNJ47965567, that antagonized the BzATP damage (Figure 6). We also examined the expression levels of TLR-4, which is a receptor linking oxidative stress and inflammatory priming in DR [72], along with the mRNA levels of IL-1β, IL-6, TNF-α, and IL-8 in endothelial cells. As expected, HG/BzATP treatment led to a significant increase in the expression levels of TLR-4 mRNA as well as all the mRNAs of the analyzed pro-inflammatory cytokines (Figure 7). These changes were paralleled by enhanced intracellular levels of ROS compared to control conditions (NG) (Figure 6). Of note, differently by JNJ47965567, the pre-treatment with DHTS significantly decreased the expression levels of TLR-4 and of IL-1β, IL-6, TNF-α, and IL-8 in endothelial cells induced by HG/BzATP treatment (Figure 7). Worthy of note, JNJ47965567 significantly decreased IL-1β, TNFα, and IL-8 expression levels, while decreasing, although not significantly, TLR-4 and IL-6 expression levels. Therefore, DHTS, bearing a multimodal activity, would affect, through other signaling pathways, the expression of TLR-4 and IL-6. These data are in accordance with a very recent publication of Yuan et al. demonstrating that DHTS exhibits an anti-inflammatory effect both in vitro and in vivo by acting on TLR-4 [73]. In line with our findings, other studies have shown the potential antioxidant and anti-inflammatory activities of both DHTS and JNJ47965567 in different experimental disease models characterized by oxidative stress and inflammation [36,74,75,76].

As mentioned before, it has been shown that P2X7R activation leads to increased ROS production in retinal micro-vessels [30] and VEGF release [77] in which the latter is able to initiate BRB breakdown in early diabetes [78]. VEGF-A is an established target and biomarker of DR [79], and DHTS has been shown to modulate TNF-α and VEGF expression by inhibiting Elav-1 (HuR) protein [35,53]. P2X7R agonist BzATP increased the expression level of VEGF-A mRNA (Figure 8). This enhanced expression was accompanied by the modulation of the expression of the other two factors strictly related to BRB damage and DR progression, Cx-43 and ICAM-1. In particular, the junctional endothelial protein Cx-43 was down-regulated by the challenge with HG/BzATP, according to other studies in which Cx-43 decreased levels are associated with the promotion of retinal vascular lesions typical of DR [80,81]. The present results are also in line with our recent findings obtained by employing an endothelial cell monolayer where the presence of BzATP significantly down-regulated the expression of Cx-43 when compared to NG conditions [32]. The pre-treatment with JNJ47965567 or DHTS led to Cx-43 expression levels to values comparable of control cells. The same protective effects were observed by the analysis of ICAM-1 expression. In fact, both JNJ47965567 and DHTS molecules were able to counteract the ICAM-1 up-regulation induced by HG/BzATP (Figure 8). This is relevant since ICAM-1 over-expression has been associated with diabetic retinal leukostasis and vascular leakage in a rat model of streptozotocin-induced diabetes [19]. Furthermore, a clinical study showed a remarkable association between the genotype distribution or the allele frequency of the ICAM-1 K469E polymorphism and the risk to develop DR in type 2 diabetes mellitus patients [82]. Other findings linked the production of VEGF with the induction of ICAM-1 and retinal leucocyte adhesion, culminating in BRB breakdown and endothelial cell injury along with promotion of neovascularization [79]. Our findings showed that DHTS is an intriguing compound with multifunctional activity because it seems to antagonize HG/BzATP stimulus. At least in our in vitro model, we can speculate that DHTS might inhibit P2X7R activity. Furthermore, we explored the expression network linked to P2X7R signaling, highlighting that modulation of P2X7R activity would impact a retinal response through several biochemical pathways, such as angiogenesis and leukostasis. Therefore, P2X7R as a pharmacological target for treatment of DR is worthy of further studies along with the pharmaceutical development of DHTS (hit compound-P2X7R antagonist) for the treatment of DR.

## 4. Materials and Methods

### 4.1. Materials and Reagents

All materials and reagents were of an analytical grade and purchased from Thermo Fisher Scientific (Waltham, MA, USA) or Sigma-Aldrich (St. Louis, MO, USA) unless specified otherwise. The three human retinal cell lines (endothelial cells, pericytes, and astrocytes) along with astrocyte cell medium (AM), pericyte cell medium (PM), endothelial cell medium (ECM), endothelial cell growth supplement (ECGS), fetal bovine serum (FBS), pericyte supplement factor (PGS), astrocytes supplement factor (AGS), polylysine (PLL), and penicillin–streptomycin were purchased from INNOPROT (Derio, Bizkaia, Spain). JNJ47965567 was purchased by Tocris Bioscience (Bristol, United Kingdom). Cell culture inserts, 75-cm^2^ polystyrene culture flasks, 12-well and 96-well plates, and rat-tail collagen type I were obtained from Corning Inc. (Corning, NY, USA). 2′,7′-dichlorofluorescin diacetate (DCFDA)—cellular ROS assay kit was purchased from Abcam (Cambridge, United Kingdom). Anti-VE-cadherin primary antibody was supplied by Cell Signaling (Leiden, Netherlands). Na-F was obtained from Santa Cruz Biotechnology, Inc. (Dallas, TX, USA). The materials necessary to perform qRT-PCR experiments (QuantiTect SYBR Green PCR Kit, RNase-free DNase Set, and QuantiTect Primer Assays) were purchased from Qiagen (Hilden, Germany), while 18S rRNA, TLR-4, P2X7R, Cx-43, and ICAM-1 primers were all purchased by Eurofins MWG Synthesis GmbH (Ebersberg, Germany).

### 4.2. Molecular Modeling and Molecular Docking

The human full-length model of P2X7R has been built with the advanced homology modeling task of Schrodinger Maestro, using the following input: primary sequence (uniprot accession# Q99572) and the template full-length rat P2X7 apo structure (PDB:6U9V). The hP2X7 model was minimized with the Prime^®^ Schrodinger Maestro task, using implicit solvation and the membrane. The protein model quality check has been carried out and the Ramachandran plot was calculated. SitMap^®^ identified three druggable pockets, the allosteric, orthosteric, and cytosolic cavities at a hP2X7 full-length model. Glide^®^ grids were built and grid origin was fixed at the center of mass of the SitMap pockets. The previously published compounds database [35] was modified including validated allosteric P2X7R agonists. The virtual screening was carried out at the allosteric P2X7R pocket, according to a previous published protocol [83]. SIFT structural interaction fingerprint analysis [84] was aimed at identification of compounds bearing binding similarity with JNJ47965567, which is a validated P2X7R allosteric antagonist. Glide^®^ docking was carried out for DHTS at allosteric, orthosteric, and cytosolic pockets. MM/GBSA calculation, using implicit solvation and the membrane, was carried out according to a previous published protocol [36].

### 4.3. Cell Culture Protocol and Treatment

A previous human triple co-culture BRB model [33] was used in the present study. Briefly, after PLL coating of plates and inserts, human retinal pericytes were seeded on the bottom side of the inserts. After an incubation step, inserts were rotated by 180° and inserted into a 12-well plate containing pericytes’ medium. Human retinal astrocytes were seeded on a 12-well plate containing astrocytes medium and left to incubate overnight. The day after, the inserts containing pericytes were moved into the 12-well plate holding astrocytes, and human retinal endothelial cells were seeded on the top side of the inserts. At this point, the three cell types were maintained in a medium consisting of a mixture of the three cell lines’ media (1:1:1). The day of the experiment, the medium was added of glucose (at the final concentration of 40 mM, indicated as HG) + BzATP (200 μM) in the absence or in the presence of JNJ47965567 (100 nM) or DHTS (500 nM) for 48 h, while the co-culture medium containing a physiological glucose concentration (5 mM, indicated as NG) was used as a control [32]. In particular, JNJ47965567 or DHTS were used as a pre-treatment of 2 h. The selection of BzATP (selective P2X7R agonist), JNJ47965567 (selective validated allosteric P2X7R antagonist), and DHTS (putative allosteric P2X7R antagonist) concentrations was made based on preliminary studies (dose response) carried out in our in vitro BRB model. BzATP was always used in combination with HG based on previous results obtained by stimulating the endothelial cell monolayer with HG, BzATP, or a combination of them (the latter being more effective) [32] as well as in preliminary experiments carried out on our BRB model.

### 4.4. BRB Integrity Assessment

The activity of DHTS and JNJ47965567 as antagonists of HG + BzATP challenge was evaluated by measurements of TEER by using a Millicel-Electrical Resistance System (ERS2) (Merck, Millipore, Burlington, MA, USA) at different time points: 0 (T0), 1 (T24), and 2 (T48) days after treatment as previously described [33,85].

To evaluate the modification of paracellular permeability under the above-mentioned conditions, the luminal-to-abluminal movements of Na-F across endothelial cell monolayers after 48 h of treatment were measured by using a Varioskan Flash microplate reader (Thermo Fisher Scientific, Waltham, MA, USA) as previously described [33].

### 4.5. Immunocytochemistry

Astrocytes, pericytes, and endothelial cells were characterized as previously described [33]. To evaluate ZO-1 expression levels, endothelial cell monolayers were washed in PBS, fixed with ice-cold acetone (−20 °C for 15 min), and incubated with ice-cold methanol (−20 °C for 20 min). Cells were then permeabilized with a solution containing PBS, NGS (5%), and Triton-X 100 (0.1%) for 10 min at RT and incubated with the ZO-1 antibody (1:100) overnight at 4 °C. After PBS washings, endothelial cells were incubated with FITC-conjugated goat anti-rabbit antibody (1:300) for 1 h at RT in the dark, while cell nuclei were marked by using DAPI (1:10000) for 10 min at RT in the dark. To assess VE-cadherin expression levels, endothelial cells were fixed in 4% PFA for 10 min at RT, permeabilized with a solution containing Triton-X 100 (0.3%) at RT for 5 min, washed three times in PBS, and blocked with BSA (1%)/PBS for 1 h at RT. Cells were then incubated with the VE-cadherin (1:100) antibody overnight at 4 °C. Next, endothelial cells were washed in PBS, incubated with FITC-conjugated goat anti-rabbit (1:300) for 1 h at RT in the dark, and incubated with DAPI for 10 min at RT in the dark.

The semi-quantitative evaluation of ZO-1 and VE-cadherin expression levels was carried out as previously described [32,33]. Coverslips were mounted on glass slides through the use of mounting medium and analyzed by using a Leica TCS SP8 confocal laser scanning microscope (Leica Biosystems, Wetzlar, Germany) or an epifluorescent Zeiss Observer Z1 microscope (Carl Zeiss Microscopy GmbH, Oberkochen, Germany). ZO-1 and VE-cadherin immunostaining images were acquired at 40× and 20× magnifications, respectively, and analyzed with ImageJ software [86]. Measurements of an average gray scale intensity were carried out at the cell-cell interface in 10 random areas of 10 image rotations [32]. A number of cells higher than 30 was analyzed for each condition.

### 4.6. Measurement of ROS Production

The ability of JNJ47965567 and DHTS in counteracting the changes in intracellular ROS due to HG + BzATP treatment for 48 h was carried out in endothelial cells, which is part of the in vitro tri-culture model, using a 2′,7′-dichlorofluorescin diacetate (DCFDA) cellular ROS assay kit, according to the manufacturer’s recommendations. To determine total ROS formation, the fluorescence (485 nm excitation/535 nm emission) was measured by using a Varioskan Flash microplate reader and normalized to the fluorescent intensity of control conditions (NG).

### 4.7. qRT-PCR

The procedure to extract the RNA by a TRIzol reagent from the endothelial cells, part of the in vitro BRB model, is the same as recently described by us [32]. RNA concentrations were determined by measuring the absorbance (260 nm) with a NanoDrop^®^ ND-1000 (Thermo Fisher Scientific) and the RNA quality was tested by a Qubit^®^ 3.0 Fluorometer (Thermo Fisher Scientific Inc. (Pittsburgh, PA, USA)). For reverse transcription, sample amplification, fluorescence data collection, and sample quantification, the same protocol as previously described was used [87,88]. The details regarding QuantiTect Primer Assays employed for the gene expression analysis is reported in Table 2.

The sequences of the primers purchased by Eurofins MWG Synthesis GmbH (Ebersberg, Germany) are the following: 18S rRNA (forward: 5′-AGT CCC TGC CCT TTG TAC ACA-3′; reverse: 5′-GAT CCG AGG GCC TCA CTA AAC-3′), TLR-4 (forward: 5′-ATA TTG ACA GGA AAC CCC ATC CA-3′; reverse: 5′-AGA GAG ATT GAG TAG GGG CAT TT-3′), P2X7R (forward: 5′-AAG CTG TAC CAG CGG AAA GA-3′; reverse: 5′-GCT CTT GGC CTT CTG TTT TG-3′), Cx-43 (forward: 5′-GAG TTT GCC TAA GGC GCT C-3′; reverse: 5′-AGG AGT TCA ATC ACT TGG CG-3′), ICAM-1 (forward: 5′-ATG CCC AGA CAT CTG TGT CC-3′; reverse: 5′-GGG GTC TCT ATG CCC AAC AA-3′), and MMP-9 (forward: 5′-CTT TGA GTC CGG TGG ACG AT-3′; reverse: 5′-TCG CCA GTA CTT CCC ATC CT-3′. The gene 18 rRNA was selected as an internal control gene to normalize the RNA levels of each gene of interest. Fold changes were calculated by the relative quantification (2^–∆∆Ct^) method.

### 4.8. Statistical Analysis

Statistical analysis was performed by using GraphPad Prism 7 (GraphPad Software, La Jolla, CA). One-way or two-way ANOVA, followed by Tukey *post-hoc* test, was used for multiple comparisons. Only two-tailed *p*-values < 0.05 were considered statistically significant. All experiments were performed at least in triplicate and reported as means ± SD.

## 5. Conclusions

The effects of the combined treatment with HG and the selective P2X7R agonist 2BzATP were investigated in our recently developed in vitro BRB model, more closely mimicking the inner retinal barrier compared to the cell monolayer, entirely based on human retinal cells. This stimulation led to significant BRB breakdown (decreased TEER and increased Na-F permeability), along with a significant reduction of both ZO-1 and VE-cadherin in endothelial cells. An increased oxidative stress, measured as a total intracellular ROS, and an up-regulation of the genes responsible for the formation of P2X7R and pro-inflammatory mediators was also observed in endothelial cells, which is part of the BRB model. The predicted P2X7R allosteric antagonist DHTS and the validated P2X7R antagonist JNJ47965567 significantly antagonized HG/BzATP-induced damage in our BRB model. In particular, DHTS maintained the integrity of the BRB and preserved the expression levels of endothelial junction proteins, ZO-1 and VE-cadherin. The increase of the expression of the TLR-4 gene as well as of IL-1β, IL-6, TNF-α, and IL-8 pro-inflammatory genes was also significantly counteracted by both compounds with the DHTS showing a greater protective effect against the HG/BzATP insult. The anti-inflammatory features along with the antioxidant action of DHTS were also demonstrated by the down-regulation of VEGF-A and ICAM-1 mRNA expression levels, the rescue of Cx-43, and the decrease in total ROS.

In conclusion, we provided new findings pointing out the therapeutic potential of DHTS in preventing and/or counteracting the blood retinal barrier dysfunctions elicited by high glucose and P2X7R activation.

## Figures and Tables

**Table 1:** Predicted score of P2X7R inhibitors binding. DHTS structural interaction fingerprints clustered with JNJ47965567, a validated P2X7R inhibitor. A438079 is another selective P2X7R inhibitor [36], that did not cluster with DHTS or JNJ47965567.

	Docking Score	ΔG_binding_ Allosteric (kcal/mol)	ΔG_binding_ Orthosteric (kcal/mol)	ΔG_binding_ Cytosolic (kcal/mol)
DHTS	−7.0	−59	−49	−30
JNJ47965567	−8.0	−86	N.A.	N.A.
A438079	−7.5	−56	N.A.	N.A.
Quercetin	−6.0	−30	−20	−30

**Table 2:** The list of primers used for qRT-PCR.

Official Name ^#^	Official Symbol	Alternative Titles/Symbols	Detected Transcript	Amplicon Length	Cat. No. ^§^
interleukin 1, beta	IL1B	IL-1, IL1F2, IL1beta, IL1-BETA	NM_000576, XM_006712496	117 bp 117 bp	QT00021385
interleukin 6	IL6	CDF, HGF, HSF, BSF2, IL-6, BSF-2, IFNB2, IFN-beta-2	NM_000600, XM_005249745	107 bp 107 bp	QT00083720
tumor necrosis factor	TNF	DIF, TNFA, TNFSF2, TNLG1F, TNF-alpha	NM_000594	98 bp	QT00029162
vascular endothelial growth factor A	VEGFA	VPF, VEGF, MVCD1	NM_001025366, NM_001025367, NM_001025368, NM_001033756, NM_001171623, NM_001171624, NM_001171625, NM_001171626, NM_001171629, NM_003376, NM_001287044	273 204 150 150 273 222 204 150 150 222 150	QT01010184
chemokine (C-X-C motif) ligand 8	CXCL8	GCP-1, GCP1, IL8, LECT, LUCT, LYNAP, MDNCF, MONAP, NAF, NAP-1, NAP1, SCYB8	NM_000584	102 bp	QT00000322
cytochrome b-245 beta chain	CYBB	CGD, NOX2, IMD34, AMCBX2, GP91-1, GP91PHOX, p91-PHOX, GP91-PHOX	NM_000397	124 bp	QT00029533
transforming growth factor beta 1	TGFB1	CED, LAP, DPD1, TGFB, IBDIMDE, TGFbeta, TGF-beta1	NM_000660	108 bp	QT00000728

^#^https://www.ncbi.nlm.nih.gov/gene/. ^§^https://www.qiagen.com/it/shop/pcr/real-time-pcr-enzymes-and-kits/two-step-qrt-pcr/quantitect-primer-assays/.
